# Development and Validation of Two Prediction Models for 72-Hour Mortality in High-Risk Trauma Patients Using a Benchmark Dataset: A Comparative Study of Logistic Regression and Neural Networks Models

**DOI:** 10.7759/cureus.40773

**Published:** 2023-06-22

**Authors:** Mehmet Muzaffer Islam

**Affiliations:** 1 Department of Emergency Medicine, Umraniye Training and Research Hospital, Istanbul, TUR

**Keywords:** comparison, logistic regression, neural networks, mortality, trauma

## Abstract

Background

Many studies have been conducted to develop scoring systems for trauma patients, with the majority using logistic regression (LR) models. Neural networks (NN), which is a machine learning algorithm, has a potential to increase the performance of these models.

Objectives

The aim of this study was to develop and validate two separate prediction models for 72-hour mortality of high-risk trauma patients using LR and NN and to compare the performances of these models in detail. We also aimed to share the SPSS calculators for our models.

Materials and methods

This is a retrospective, single-center study conducted using a benchmark dataset where the patients were retrospectively gathered from a level 1 trauma center. Patients older than 18 years of age, who had multiple injuries, and were treated at the University Hospital Zurich between January 1, 1996, and January 1, 2013, were included. Patients with a condition that may have an impact on the musculoskeletal system, with Injury Severity Score<16, and with missing outcome data were excluded.

Results

A total of 3,075 patients were included in the analysis. The area under the curve values of the LR and NN models for predicting 72-hour mortality in patients with high-risk trauma in the hold-out cohort were 0.859 (95% CI=0.836 to 0.883) and 0.856 (95% CI=0.831 to 0.880), respectively. There was no statistically significant difference in the performance of the models (p = 0.554, DeLong's test).

Conclusion

Both of the models showed good discrimination. Our study suggests that the NN and LR models we developed hold promise as screening tools for predicting 72-hour mortality in high-risk trauma patients. These models were made available to clinicians as clinical prediction tools via SPSS calculators. However, further external validation studies in diverse populations are necessary to substantiate their clinical utility. Moreover, in subsequent studies, it would be beneficial to derive NN models with substantial events per predictor variable to attain more robust and greater predictive accuracy. If the dataset is relatively limited, using LR seems to be a viable alternative.

## Introduction

Trauma is a significant cause of mortality worldwide, accounting for more than four million deaths each year [1]. Identifying high-risk trauma patients early and accurately predicting their mortality is crucial for timely and appropriate medical interventions to improve patient outcomes [2]. Many studies have been conducted to develop scoring systems for trauma patients, with the majority using logistic regression (LR) models [3-5]. While LR models have been effective in predicting mortality, they may not define complex and nonlinear relationships between predictors and outcomes. In recent years, machine learning (ML) algorithms, such as neural networks (NN), have become increasingly popular in medical research for their ability to model complex and nonlinear relationships [6]. NN can process large amounts of data and identify patterns that may not be apparent using traditional statistical methods. Thus, they have a potential to improve the accuracy of the model.

In a 2019 meta-analysis comparing LR and NN models in predicting various outcomes in trauma patients, the area under the curve (AUC) of the NN model was calculated as 0.91 (95% CI = 0.89 to 0.93), and the AUC of the LR model was 0.89 (95% CI = 0.87 to 0.90) [7]. Similar results were obtained in systematic reviews conducted for other patient groups. However, the authors stated that although NN performance was often superior to LR, the difference in performance was not clinically significant [8]. Both methods possess inherent strengths and limitations with respect to their applicability in medical decision-making processes.

The primary outcome of this study is to develop and validate two separate prediction models for 72-hour mortality of high-risk trauma patients using LR and NN and to compare and discuss the performances of these models in detail. We also aimed to share the SPSS calculators (available in the Appendix in xml format) for our models to allow researchers to perform future validation studies.

## Materials and methods

We conducted this retrospective cohort study using the benchmark dataset accessed from the Dryad database [9,10]. Dryad serves as an open source data platform and emphasizes the principles of open availability and widespread reuse of research data across diverse disciplines. The approval of the local review board (Umraniye Egitim ve Arastirma Hastanesi Etik Kurulu) was obtained. The approval was dated March 22, 2023, and the study was assigned the protocol number E-54132726-000-211921076/211921076. This study was designed and written in accordance with the “Transparent Reporting of a multivariable prediction model for Individual Prognosis Or Diagnosis (TRIPOD)” statement [11].

Definitions and selection of the participants

We defined “high-risk” trauma patients as having an Injury Severity Score (ISS) of 16 or greater according to the 2016 study of Newgard et al. [2].

The original study where the benchmark dataset was obtained included trauma patients older than 18 years of age, who were treated at the University Hospital Zurich between January 1, 1996, and January 1, 2013. Patients with chronic illnesses and oncological conditions or genetic abnormalities that may have an impact on the musculoskeletal system were stated to be excluded [10].

Since we conducted this study on high-risk trauma patients, we excluded patients who did not meet this definition (patients with ISS<16). Moreover, in accordance with a previous study using this benchmark dataset, we also excluded patients with missing outcome information or miscoded data [12].

Study protocol

**Figure 1:**
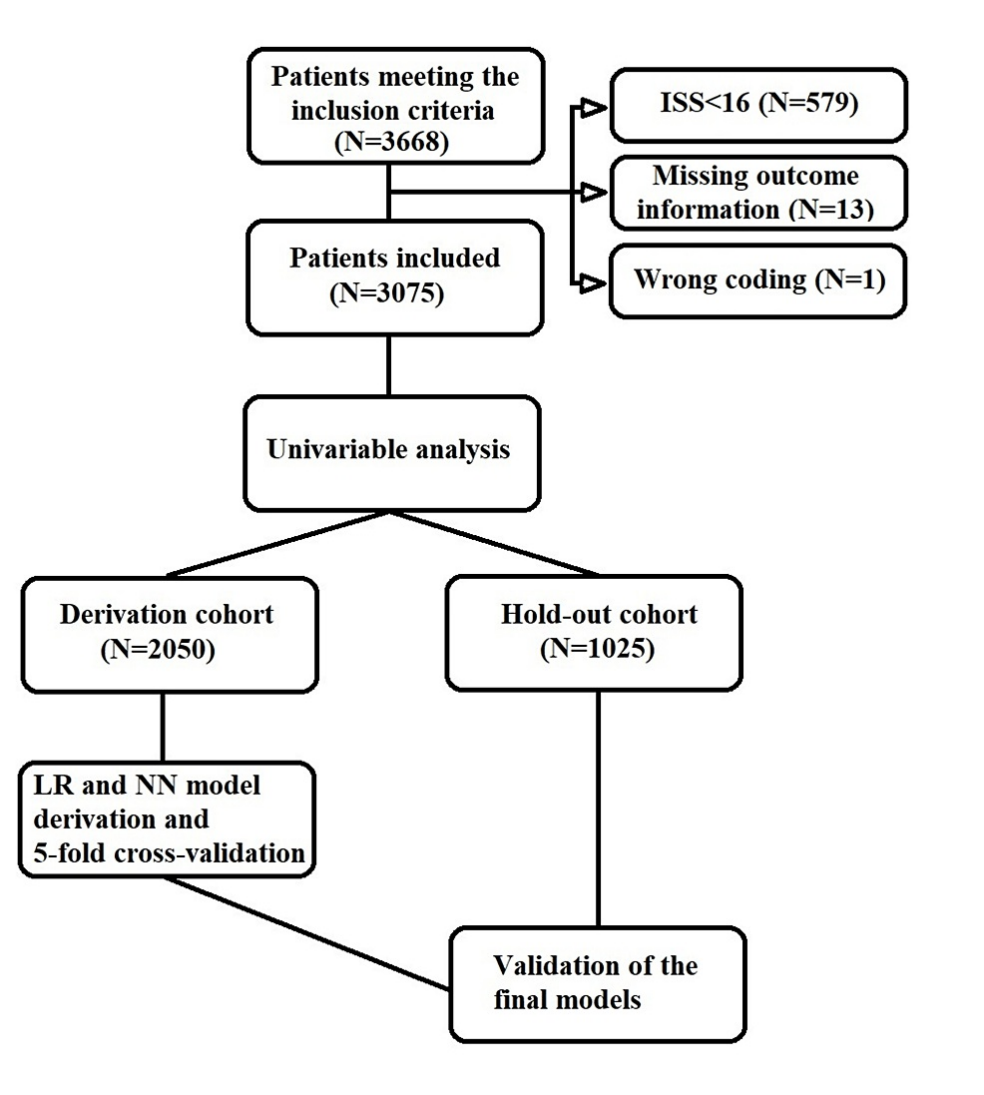
Patient flowchart and the summary of the study protocol. ISS, Injury Severity Score; LR, logistic regression; NN, neural networks

Following the application of the inclusion and exclusion criteria, we performed a multiple imputation procedure to handle the missing data. The medians of the pre- and post-imputation datasets were summarized. The imputed dataset was subjected to univariate analysis in terms of 72-hour mortality. Following this analysis, the dataset was randomly divided into derivation and hold-out cohorts at a 3:1 ratio with the "random split-sample development and validation" method outlined in the TRIPOD statement [11]. To accomplish this, we used an online randomizer [13]. Subsequently, we derived LR and NN models, conducted a five-fold cross-validation for internal validation, and presented the results. Finally, we externally validated our models in the hold-out cohort. The SPSS calculators of the LR and NN models are provided in the Appendix. A summary of the study protocol is provided in Figure 1.

Missing data

Missing data were imputed using “multiple imputations.” This process involves creating multiple plausible imputations for each missing value, which results in a more accurate estimate of the missing data and reduces bias in the analysis. Due to the high missing rate in the body mass index variable (47.4%), it was excluded from the imputation process. After the procedure, the analysis was performed on the complete dataset containing the imputed values.

Sample size calculation

The estimated mortality prevalence of patients with polytrauma is reported to be 20% to 25% in the literature [14]. Based on the study by Peduzzi et al., assuming a significance level of α = 0.05, a power of β = 0.80, a prevalence of 25%, an odds ratio of 2.0 as the expected effect size, and 10 potential predictors in the model, we needed at least 365 patients to detect a significant effect of the new model on mortality with a power of 0.80 and a significance level of 0.05 [15]. Additionally, the study also suggested that there should be a minimum of 10 events per potential predictor in the dataset [15]. It was determined that the benchmark dataset was of sufficient size to meet these requirements.

Statistical analysis

Data analysis and modeling were performed using IBM SPSS Statistics version 29 (IBM Corp., Armonk, NY, USA). Since none of the continuous data were distributed normally, median values with 25-75% quartile ranges were presented, and the Mann-Whitney U test was used to compare groups. Categorical data were presented as frequencies and percentages, and group comparisons were performed using the chi-square test. To evaluate the diagnostic performance of the models, we used receiver operating characteristics (ROC) and determined the optimal cut-off points with Youden’s index. For comparing two AUC values, we used DeLong’s test. We set the statistical significance level at p<0.05.

We used variables that demonstrated a significant difference between mortality groups in the univariate analysis as predictors in both LR and NN models. We used binary logistics and forced-entry method to develop the LR model. We assessed the goodness of fit using the Hosmer-Lemeshow test and checked for multicollinearity by examining the correlation matrix for any strong correlations between the predictors. We examined Cook's distances for all outliers to determine their effect on the model. The overall performance of the model was evaluated using Nagelkerke R-squared. To identify the most valuable predictor for our model, we examined Wald statistics.

For our NN model, we used a multilayer perceptron with two hidden layers. The first hidden layer had four neurons, and the second layer had three neurons. We used hyperbolic tangent as the activation function for the hidden layers, softmax as the activation function for the output layer, and cross-entropy as the output error function. Because Adam, which is the most commonly preferred optimization method, was not available in the SPSS, we used gradient descent as the optimization algorithm with a learning rate of 0.04 and momentum of 0.9.

Outcome measures

The primary outcome of this study is to develop and validate two separate prediction models for 72-hour mortality of high-risk trauma patients using LR and NN and to compare the performances of these models. We also aimed to share the SPSS calculators (available in the Appendix in xml format) for our models to allow researchers to perform future validation studies.

## Results

Missing data, basic characteristics, and univariate analysis

The benchmark dataset contained 3,668 patients. We excluded 579 patients with ISS<16, 13 patients with missing outcome data, and one patient with invalid body mass index coding. After applying all the exclusion criteria, a total of 3,075 patients were enrolled in our study (Figure 1). Missing data were handled through multiple imputations. Body mass index variable had a total of 1,459 (47.4%) missing values and was not imputed or used as a predictor of imputation. After the imputation process, the median age of the patients was 41 (29 to 50) years, and 1,775 (73.6%) of the patients were male. Within the total study cohort, 687 (22.3%) patients had the primary outcome of in-hospital mortality within 72 hours. Table 1 presents a summary of the imputation process and the basic characteristics of the patients before and after the multiple imputations.

**Table 1:** Summary of the multiple imputation process and the basic characteristics of the patients before and after multiple imputations (N=3,075). BMI, body mass index; GCS, Glasgow Coma Scale; ISS, Injury Severity Score

	Cases missing, N (%)	Before imputation, median (25-75% quartiles) or N (%)	After imputation, median (25-75% quartiles) or N (%)
Age	0 (0%)	43 (28 to 61)	43 (28 to 61)
Sex (male)	0 (0%)	1775 (73.6%)	1775 (73.6%)
BMI	1459 (47.4%)	25 (22 to 27)	Not performed
GCS	42 (1.4%)	6 (3 to 15)	6 (3 to 15)
ISS	1 (<0.1%)	27 (22 to 38)	27 (22 to 38)
pH	833 (27.1%)	7.33 (7.26 to 7.38)	7.33 (7.26 to 7.39)
Base excess (mmol/L)	704 (22.9%)	-3 (-6 to -0.8)	-3.1 (-6.3 to -0.7)
Lactate (mmol/L)	473 (15.4%)	2.3 (1.4 to 3.6)	2.3 (1.4 to 3.8)
72-hour mortality	0 (0%)	687 (22.3%)	687 (22.3%)

In the univariate analysis performed after the multiple imputation, age, ISS, and lactate levels were found to be significantly higher in the mortality group (p<0.001 for all). Whereas, GCS, base excess, and pH were significantly lower (p<0.001 for all). There was no significant difference between the mortality groups in terms of sex (p=0.084). The univariate analysis is summarized in Table 2.

**Table 2:** Descriptives of the study population and the comparison of the 72-hour mortality groups. BMI, body mass index; GCS, Glasgow coma scale; ISS, Injury severity scale

	Survivors, median (25-75% quartiles) or N (%)	Mortality (+), median (25-75% quartiles) or N (%)	p-Value
Age	42 (27 to 58)	51 (32 to 73)	<0.001
Sex (male)	1775 (74.3)	488 (71)	0.084
BMI (N=1,616)	25 (22 to 27)	25 (22 to 28)	0.425
GCS	12 (3 to 15)	3 (3 to 3)	<0.001
ISS	26 (20 to 34)	34 (25 to 50)	<0.001
pH	7.34 (7.28 to 7.39)	7.27 (7.16 to 7.36)	<0.001
Base excess (mmol/L)	-2.6 (-5.3 to -0.5)	-5.9 (-10.5 to -2.3)	<0.001
Lactate (mmol/L)	2.1 (1.3 to 3.3)	3.7 (2.2 to 6.3)	<0.001

Derivation and the internal validation of the final models

The dataset was divided randomly into a derivation cohort and a hold-out cohort using a 3:1 ratio, resulting in 2,050 patients in the derivation cohort and 1,025 patients in the hold-out cohort. The models were derived in the derivation cohort and then validated in the hold-out cohort (Figure 1).

We used the forced entry method in the LR model and included ISS, Glasgow coma scale (GCS), pH, base excess, lactate, and age as predictors. The assumption of goodness of fit was met (p=0.113, Hosmer-Lemeshow test), indicating that the model fit the data well. There were no strong correlations between the predictors; therefore, no multicollinearity was detected. The final LR model correctly classified 84% of the patients and explained 46.2% of the variance in the derivation cohort (Nagelkerke R-squared = 0.462). GCS, age, ISS, and lactate were identified as independent predictors of 72-hour mortality in patients with high-risk trauma (p<0.001 for all). The most valuable predictor was GCS, with a Wald statistics of 175.2. Although there were 13 outlier patients, none of their Cook's distances exceeded 1, and they were not excluded from the model. Table 3 summarizes the regression model and the regression function.

**Table 3:** Summary of the logistic regression model. Regression function = -2.899 + (GCS * -0.228) + (Age * 0.031) + (ISS * 0.040) + (Lactate * 0.216) + (Base excess * -0.043) + (pH * -0.091) GCS, Glasgow Coma Scale; ISS, Injury Severity Score

	β coefficient	Wald statistics	p-Value	OR (95% CI)
GCS	-0.228	175.2	<0.001	0.796 (0.770 to 0.824)
Age	0.031	89.5	<0.001	1.032 (1.025 to 1.039)
ISS	0.040	73.3	<0.001	1.041 (1.031 to 1.050)
Lactate	0.216	32.9	<0.001	1.241 (1.153 to 1.336)
Base excess	-0.043	3.4	0.065	0.958 (0.915 to 1.003)
pH	-0.091	0.02	0.899	0.913 (0.223 to 3.732)
Constant	-2.899	0.3	0.586	NA

**Figure 2:**
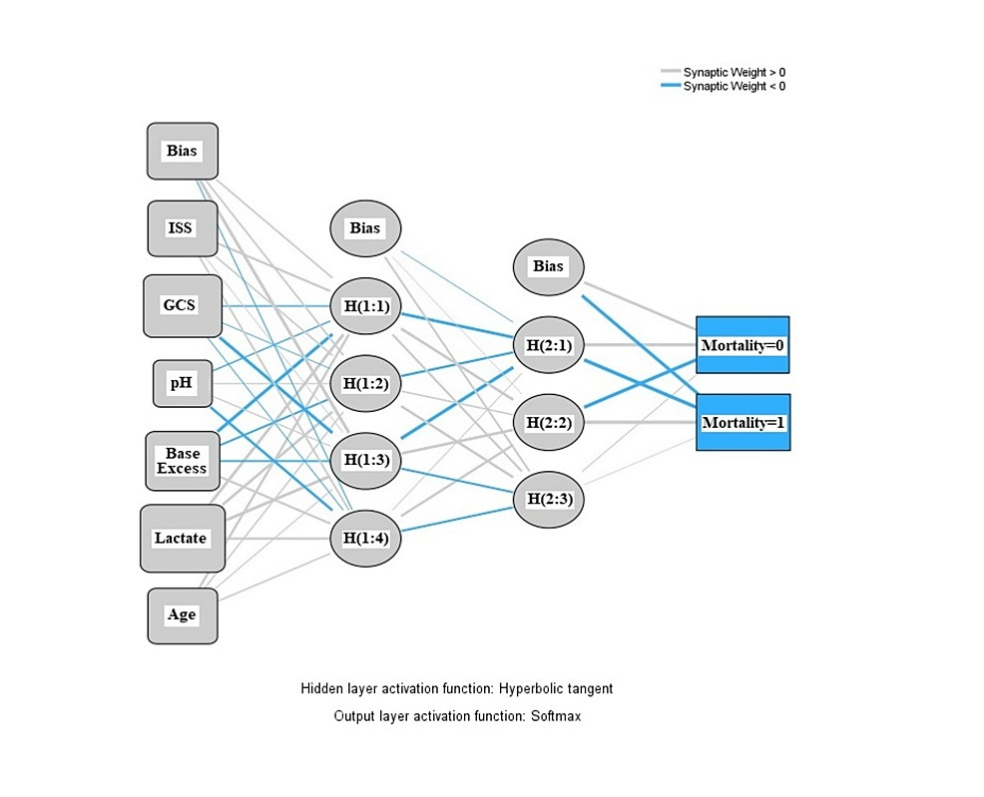
Synaptic weights of the neural networks model. GCS, Glasgow Coma Scale; ISS, Injury Severity Score

**Figure 3:**
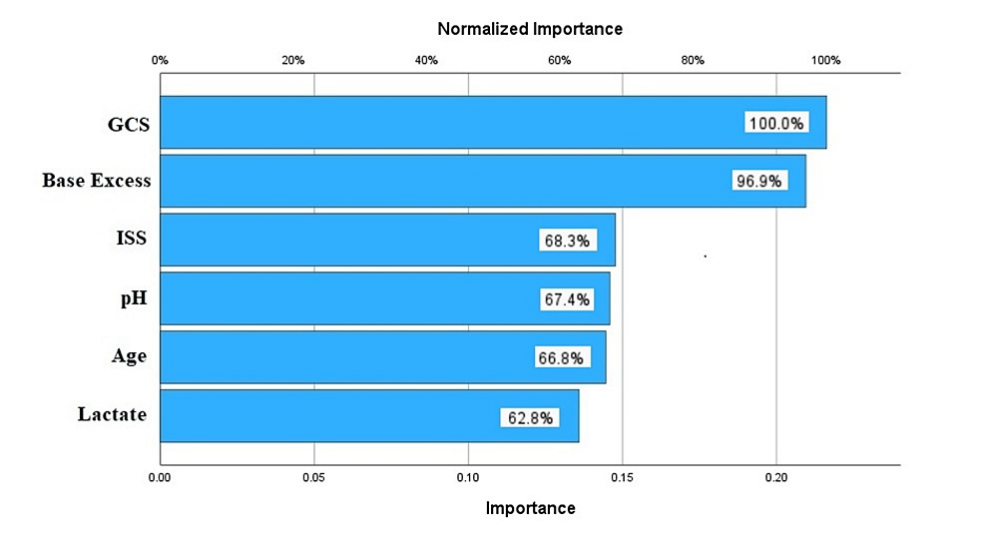
Importance chart of the predictors in the neural networks model. GCS, Glasgow Coma Scale; ISS, Injury Severity Score

For the NN model, multilayer perceptron was used, and ISS, GCS, pH, base excess, lactate, and age were included as the predictors. After the optimization with gradient descent, the final model was able to classify 84% of the patients correctly in the derivation cohort. The NN model was described, and the synaptic weights are shown in Figure 2. The most valuable predictors in the NN model were GCS and base excess. The normalized importance graphics is summarized in Figure 3.

The mean AUC of the LR and NN models in the derivation cohort were 0.873 (95% CI= 0.862 to 0.884) and 0.869 (95% CI= 0.858 to 0.880) respectively. In the validation cohort, the mean AUC for the LR and NN models were 0.868 (95% CI= 0.828 to 0.908) and 0.868 (95% CI= 0.837 to 0.900), respectively. Table 4 summarizes the performance results of the models using five-fold cross-validation.

**Table 4:** The comparison of the five-fold cross-validation results of the logistic regression and the neural networks models. AUC, area under the curve

		Training				Testing			
		AUC	Sensitivity	Specificity	Accuracy	AUC	Sensitivity	Specificity	Accuracy
Logistic regression	Fold 1	0.879 (0.861 to 0.897)	48.1% (42.9 to 53.3)	94.3% (92.8 to 95.5)	83.9% (82.1 to 85.7)	0.840 (0.798 to 0.883)	39.1% (29.1 to 49.9)	94.7% (91.6 to 96.9)	82.2% (78.2 to 85.8)
	Fold 2	0.880 (0.862 to 0.898)	50% (44.8 to 55.2)	94.3% (92.8 to 95.5)	84.4 (82.5 to 86.1)	0.839 (0.797 to 0.881)	40.2% (30.1 to 51)	92.8% (89.4 to 95.4)	81% (76.9 to 84.7)
	Fold 3	0.873 (0.855 to 0.892)	48.4% (43.1 to 53.6)	95.1% (93.8 to 96.3)	84.7% (82.9 to 86.4)	0.868 (0.831 to 0.905)	41.3% (31.3 to 52.1)	93.7% (90.5 to 96.1)	82% (77.9 to 85.6)
	Fold 4	0.873 (0.854 to 0.892)	46.9% (41.7 to 52.1)	94.7% (93.3 to 95.8)	83.9% (82.1 to 85.7)	0.876 (0.840 to 0.912)	46.2% (35.6 to 56.9)	95.9% (93.1 to 97.8)	84.8% (81 to 88.2)
	Fold 5	0.858 (0.839 to 0.878)	42.5% (37.4 to 47.7)	94.5% (93.1 to 95.7)	82.9% (81 to 84.7)	0.918 (0.889 to 0.948)	63.7% (53 to 73.7)	93.4% (90.1 to 95.9)	86.8% (83.1 to 89.9)
	Mean	0.873 (0.862 to 0.884)	47.2% (43.7 to 50.7)	94.6% (94.2 to 95)	84% (83.1 to 84.8)	0.868 (0.828 to 0.908)	46.1% (33.4 to 58.8)	94.1% (92.6 to 95.6)	83.4% (80.4 to 86.3)
Neural networks	Fold 1	0.877 (0.859 to 0.896)	52.2% (46.9 to 57.4)	92.9% (91.3 to 94.2)	83.8% (81.9 to 85.5)	0.845 (0.803 to 0.887)	44.6% (34.2 to 55.3)	93.1% (89.7 to 95.6)	82.2% (78.2 to 85.8)
	Fold 2	0.879 (0.861 to 0.897)	47.8% (42.6 to 53.1)	95.7% (94.4 to 96.7)	85% (83.2 to 86.7)	0.845 (0.805 to 0.886)	40.2% (30.1 to 51)	95.3 (92.4 to 97.3)	83% (79 to 86.5)
	Fold 3	0.861 (0.841 to 0.881)	47.5% (42.3 to 52.8)	93.3 (91.7 to 94.6)	83.1% (81.1 to 84.8)	0.866 (0.828 to 0.904)	43.5% (33.2 to 54.2)	92.5% (89 to 95.1)	81.5 (77.4 to 85.1)
	Fold 4	0.870 (0.851 to 0.889)	50.7% (45.4 to 55.9)	92.1% (90.5 to 93.5)	82.8% (80.9 to 84.6)	0.883 (0.848 to 0.918)	52.8% (42 to 63.3)	93.7% (90.5 to 96.1)	84.6% (80.7 to 88)
	Fold 5	0.859 (0.840 to 0.879)	45.8% (40.6 to 51)	93.3% (91.7 to 94.6)	82.6% (80.7 to 84.4)	0.904 (0.873 to 0.934)	50.6% (39.9 to 61.2)	93.7% (90.5 to 96.1)	84.1% (80.2 to 87.5)
	Mean	0.869 (0.858 to 0.880)	48.8%(45.6 to 52)	93.5% (91.8 to 95.1)	83.5% (82.3 to 84.7)	0.868 (0.837 to 0.900)	46.3% (39.9 to 52.8)	93.7% (92.4 to 95)	83.1% (81.5to 84.7)

External validation and the comparison of the performances of the models

**Figure 4:**
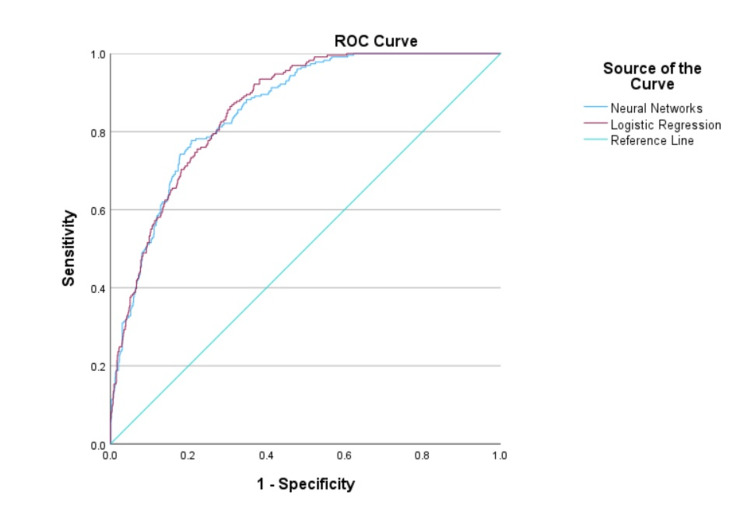
Comparison of the area under the curves of the logistic regression and neural networks models. ROC, receiver operating characteristics

The AUC values of the LR and NN models for predicting 72-hour mortality in patients with high-risk trauma in the hold-out cohort were 0.859 (95% CI: 0.836 to 0.883) and 0.856 (95% CI: 0.831 to 0.880), respectively (Figure 4). There was no statistically significant difference in the performance of the models, with a difference in AUC of 0.004 (95% CI: -0.009 to 0.017; p = 0.554, DeLong's test). The sensitivity of the LR model was 82.5% (95% CI: 77.0 to 87.2), while the sensitivity of the NN model was 78.2% (95% CI: 72.3 to 83.3). The performance metrics of the models are presented in Table 5.

**Table 5:** External validation performances of the models in the hold-out cohort.

	Logistic regression	Neural networks
AUC	0.859 (0.836 to 0.883)	0.856 (0.831 to 0.880)
Sensitivity	82.5% (77 to 87.2)	78.2% (72.3 to 83.3)
Specificity	71.6% (68.3 to 74.7)	76.4% (73.3 to 79.3)
Positive predictive value	45.5% (42.5 to 48.7)	48.8% (45.2 to 52.3)
Negative predictive value	93.4% (91.5 to 95)	92.4 (90.5 to 94)
Positive likelihood ratio	2.91 (2.57 to 3.3)	3.31 (2.87 to 3.82)
Negative likelihood ratio	0.24 (0.18 to 0.32)	0.29 (0.23 to 0.37)
Accuracy	74.1% (71.3 to 76.7)	76.8% (74.1 to 79.3)

## Discussion

Our LR and NN models demonstrated persistent results in the five-fold cross-validation. Furthermore, it is important to note that the NN model showed highly consistent results in the internal validation, with almost no performance degradation during the training and testing process. Therefore, we can safely assert that there was no significant risk of overfitting in either of the models.

Upon evaluating the models' performances on the hold-out cohort, which they had not encountered before, we observed that both models exhibited good discrimination. There was no significant difference in the performances of the models on the hold-out cohort. The LR model demonstrated relatively higher sensitivity, whereas the NN had slightly higher specificity. The performance of both models is satisfactory for use as a screening tool for high-risk trauma patients to predict 72-hour mortality by using only six simple and easy-to-acquire variables.

We observed that the order of importance of predictors differed between the models. However, the GCS remained the most valuable predictor in both models. Furthermore, the ISS was identified as the third most valuable predictor in both models.

The GCS has been extensively studied as a predictor of mortality in trauma patients, irrespective of the presence of traumatic brain injury (TBI). A number of studies have demonstrated that lower GCS scores are strongly associated with an increased risk of mortality in trauma patients [3-5]. However, while the GCS remains an important tool for assessing neurological function in trauma patients, it should be used in conjunction with other clinical and demographic variables to develop more accurate predictive models.

A higher ISS has been consistently associated with increased risk of mortality in trauma patients and is widely used as a tool for clinical decision-making in trauma centers and emergency departments [2]. In addition, ISS has been shown to be a useful predictor of other outcomes, such as length of hospital stay, need for intensive care unit admission, and development of complications [16]. While ISS is a valuable tool for assessing trauma severity and predicting mortality risk, it has some limitations that should be considered.

TBI is a leading cause of morbidity and mortality in trauma patients, and accurate prediction of TBI-related mortality risk is crucial for guiding clinical decision-making. ISS may not accurately reflect the severity of TBI, as it does not take into account important factors [17].

Hence, incorporating the GCS (or other clinical tools for assessing mental status) along with the ISS appears to be a justifiable approach to enhance the performance of a trauma model for predicting mortality, as the limitations inherent in each predictor may be offset by the strengths of the other.

LR has become the gold standard for developing clinical risk scoring systems. However, in recent years, ML algorithms, particularly NN, have gained significant popularity for binary classification problems and have been shown to yield consistent results [8]. While the ML techniques have been shown to provide consistent results in solving many classification problems, they may not offer a significant advantage over traditional methods in solving medical problems due to certain limitations. In an important systematic review published in 2019, it was noted that there is insufficient evidence to suggest that ML algorithms, including NN, yield better AUC results compared to models created with LR [18]. This review also emphasizes the low number of event per predictor variable (EPV) in existing studies, inadequacies in reporting, and the lack of use of appropriate validation techniques [18].

In another systematic review that evaluated the data of 374,365 patients, the performance of LR and ML methods in predicting mortality and readmission rates in patients with myocardial infarction was compared. Although the ML methods were found to have higher AUC values than LR, it is reported that there were deficiencies in internal validation in ML studies. It was also emphasized that most of these studies also lacked external validation [19]. Similar to the authors of this review, we attribute this situation to the lack of an accepted guideline for ML models, such as the TRIPOD statement, which has hindered the establishment of standard procedures.

LR is a theory-driven method that relies on several assumptions such as linearity, goodness of fit, and the absence of multicollinearity. The validity of these assumptions can and often should be positively influenced by the interventions of researchers during the modeling process [18]. It has been reported that LR will typically provide consistent and robust results and may even outperform ML algorithms in cases where the relationship between predictors and outcomes is linear and homogeneous [6,20].

NN, on the other hand, is purely data-driven and performs many interventions, or penalizations, required in LR automatically. When the sample size is larger, it can handle greater numbers of predictors better than LR without overfitting the model [21]. Thus, NN algorithms are reported to perform better than LR in cases where the necessary assumptions for LR are not met, the predictors are highly correlated, or the relationship between the outcome and the predictors is rather sophisticated [6,20,22].

However, ML algorithms such as NN have their unique limitations. For example, in an important study conducted in 2014, the optimism of the random forest (RF) algorithm was found to be quite high even at high EPVs (such as EPV=200), and it was reported that it usually generates worse results in the validation studies. In the same study, it was reported that LR has a reasonable optimism of around EPV=10 and even lower optimism when EPV=20-50. Therefore, it gives more robust results in studies where the dataset is relatively limited [23]. Regarding NN, it has been reported that although it requires more events compared to LR, it gives significantly consistent results at high EPVs compared to RF, and its optimism converges to zero. When the results of the aforementioned study are examined, it can be seen that the NN model needs approximately 200-250 EPVs to reach the optimism level of the LR model [23]. Considering that many ML studies in the medical literature do not provide these numbers of EPV in their study samples, it can be said that ML algorithms often fail to reach their full potential.

Furthermore, unlike LR models that can be easily calculated using values such as β coefficients and intercept, NN models require a calculator for prediction. Furthermore, unlike LR models that can be easily calculated using values such as β coefficients and intercept, NN models require a calculator for prediction, as ML models usually produce complex algorithms with low interpretability and explainability [8]. Despite this fact, in many modelling studies using ML algorithms, the models or the pragmatic calculators of the models are not usually provided, which limits researchers' ability to reproduce, validate, and compare these models effectively in future studies.

When designing this study, we followed the TRIPOD statement for both models and applied an internal validation technique in the NN model, as the TRIPOD statement suggested for LR. We reported the modeling steps in detail and shared our final models as a pragmatic SPSS calculator in the Appendix so that validation studies can be carried out. Moreover, in the derivation cohort of our study, the number of events was 458, and EPV was 76 for both models, and we observed that the models provided similar results. We believe that these aspects of our study do strongly address many of the limitations in the literature we discussed earlier.

Our study has a number of limitations that should be acknowledged. First, the data were collected and reviewed retrospectively, which may raise concerns about data security. Second, the data were obtained from patients treated approximately over a decade ago, and advances in trauma management since then may have a negative impact on the generalizability of our findings. Furthermore, while our external validation showed no significant decrease in performance with EPV = 76, we acknowledge that better model performance and smaller optimism may have been achieved with EPV values in the range of 200 to 250 in the NN model.

## Conclusions

In conclusion, our study suggests that the NN and LR models we developed hold promise as screening tools for predicting 72-hour mortality in high-risk trauma patients. These models were made available to clinicians as clinical prediction tools via SPSS calculators. However, further external validation studies in diverse populations are necessary to substantiate their clinical utility. Moreover, in subsequent studies, it would be beneficial to derive NN models with substantial events per predictor variable and larger sample sizes to attain more robust and greater predictive accuracy. If the dataset is relatively limited, using LR seems to be a viable alternative.

## Appendices

README

The variable names in your dataset should be as follows in order for this SPSS calculator to work:

1. ISS: Injury severity index on admission

2. GCS_admission: Glasgow coma scale on admission

3. pH_admission: pH value on admission

4. lct_admission: Lactate level on admission (mmol/L)

5. BE_admission: Base excess on admission (mmol/L)

6. age: Age on admission (years)

7. MORTALITY_72Hours: 72 hours mortality (0=Alive / 1=Dead)

- To use the desired model (logistic regression or neural networks), you can open SPSS: Utilities : Scoring Wizard and select the desired model’s xml file.

- Match the “dataset fields” boxes with “model fields” boxes correctly.

- Select the “probability of the selected category” as “1.”

-Click on “finish.”

 Important Note:

- After obtaining the dataset from the Dryad databank website for our study, the authors made the decision to impose an indefinite embargo on dataset access. It is crucial to emphasize that the dataset was legally acquired prior to the implementation of this embargo, and we do not perceive this situation as a violation of any terms of agreement. Should individuals be interested in accessing the original dataset used in our study, they can easily reach out to us for assistance, and we would provide them with the raw dataset with the Dryad's permission.
